# A Simple and Reliable Single Tube Septuple PCR Assay for Simultaneous Identification of Seven Meat Species

**DOI:** 10.3390/foods10051083

**Published:** 2021-05-13

**Authors:** Zhendong Cai, Song Zhou, Qianqian Liu, Hui Ma, Xinyi Yuan, Jiaqi Gao, Jinxuan Cao, Daodong Pan

**Affiliations:** 1 State Key Laboratory for Managing Biotic and Chemical Threats to the Quality and Safety of Agro-Products, Ningbo University, Ningbo 315211, China; zhendongcai@hotmail.com (Z.C.); zhousongluchen@163.com (S.Z.); m17805843103@163.com (H.M.); yxy13065810608@163.com (X.Y.); caidan1389@126.com (J.G.); caojinxuan@nbu.edu.cn (J.C.); 2 Key Laboratory of Animal Protein Deep Processing Technology of Zhejiang Province, College of Food and Pharmaceutical Sciences, Ningbo University, Ningbo 315800, China; 3 Institute of Environmental Research at Greater Bay Area, Guangzhou University, Guangzhou 510006, China

**Keywords:** septuple PCR, adulteration, meat species, mitochondrial genes, multiplex PCR

## Abstract

Multiplex PCR methods have been frequently used for authentication of meat product adulteration. Through screening of new species-specific primers designed based on the mitochondrial DNA sequences, a septuple PCR method is ultimately developed and optimized to simultaneously detect seven species including turkey (110 bp), goose (194 bp), pig (254 bp), sheep (329 bp), beef (473 bp), chicken (612 bp) and duck (718 bp) in one reaction. The proposed method has been validated to be specific, sensitive, robust and inexpensive. Taken together, the developed septuple PCR assay is reliable and efficient, not only to authenticate animal species in commercial meat products, but also easily feasible in a general laboratory without special infrastructures.

## 1. Introduction

Meat authentication is an important concern to protect consumers from illegal and unwanted ingredients [1,2,3,4]. However, meat adulteration such as unlisted, mislabeled or fraudulent ingredients has frequently been reported around the world and has become a severe global issue [4,5]. Although some laws have been enacted for ensuring the quality and safety of meat products, adulteration is still widespread due to the purpose of economic pursuit [1,6]. Poultry meat (chicken, duck and goose), especially, is frequently adulterated to red meat due to their low cost of production in Chinese markets [1,7]. As is known, soy allergy has become an arising public health concern regarding food allergies, as a small amount of soybean may elicit allergic reactions in both children and adults [8]. Notably, there is increasing evidence supporting that meat may trigger allergic reactions, especially for sensitized patients, which may cause a severe health risk of infectious diseases, metabolic disorders and allergies [9]. In addition, meat adulteration could also violate religious concerns; as is known, meat products containing pork ingredients are not permitted by Kosher and Halal food laws [10,11]. Therefore, it remains a pressing need for identifying meat species with 100% accuracy in real-world foodstuffs.

In recent years, the techniques for authenticating meat species have been continuously evolving. Many analytical methods of biological, immunological, physical, chemical, anatomical and histological analyses have been developed [2,3,4,7,12]. Among them, protein-based methods are widely used to identify meat species in composite mixtures [5,13]. Proteins are likely to be degraded, denatured or damaged in processed meat products, limiting the accuracy of the identification of meat species in thermally treated foods [13,14]. In comparison, DNA-based analytical methods coupled with polymerase chain reaction (PCR) present a reliable alternative to protein-based methods in the discrimination and mislabeling detection of meat species, as DNA molecules possess high stability and are present in every type of cell [4,5]. Both multiplex and real-time PCR techniques are highly specific and efficient in the identification of meat adulteration [15]. Real-time PCR techniques are widely used to quantify the amount of a target sequence in a reaction system. The levels of PCR amplification are monitored in real time, once per cycle, by measuring specific fluorescence signals, whose intensities reflect the amount of PCR product [16]. With the progress of molecular biology in recent years, multiplex real-time PCR techniques depending on melting curve analysis have been developed and widely adopted in the identification of meat species, and they have characteristics such as time saving, high specificity and high sensitivity [17,18,19,20]. Collectively, real-time PCR analysis shows more detailed information with regard to the identification and quantification of meat species. However, accurate quantification can only be achieved with a proper reference material because the matrix may interfere with the amplification process [21], indicating that quantification of meat fractions in real-world foodstuffs is difficult, and the results may not truly correlate to the recipe of the meat products. In comparison, multiplex PCR assay can be easily implemented with minimum effort, but much gain, to verify the identification of meat species. Recently, many studies have also constructed multiplex PCR with electrophoresis analysis to authenticate meat species with satisfying results [1]. Although much is known about multiplex PCR as duplex, triplex, tetraplex, pentaplex (quintuple) and hexaplex (sextuple) PCRs, little information is available on a multiplex PCR authenticating more than six animal species simultaneously.

Mitochondrial DNA possesses high copy numbers per cell and strong stability, which ensures a low limit of detection and its availability in both raw and cooked meat products, and it has been broadly adopted for PCR protocols [22,23]. For example, cytochrome *b* gene, D-loop, 12S and 16S rRNA genes, ATPase subunits 8 and 6 genes, and NADH dehydrogenase genes are common targets for identifying meat species [21,24,25]. All data provide reliable evidence for the roles of mitochondrial DNA sequences in animal species identification. Using mitochondrial DNA sequences obtained from turkey, goose, pig, sheep, beef, chicken and duck, we designed a set of primer pairs that specifically amplified for seven species with differential lengths through PCR assays. We next performed the specific, sensitive and cost-effective detection of the indicated primers through simplex and multiplex PCR assays. Through screening, this study develops a septuple PCR assay for identifying seven ingredients of turkey, goose, pig, sheep, beef, chicken and duck simultaneously in commercial foodstuffs.

## 2. Materials and Methods

### 2.1. Preparation of Meat Samples and DNA Extraction

Fresh meat samples of turkey, goose, pig, sheep, beef, chicken and duck were purchased from local retailers and markets and transported on ice to the laboratory for immediate processing. Samples were stored at −20 °C to prevent DNA degradation. Total DNA was extracted from various meat samples using the EasyPure^®^ Genomic DNA Kit (Beijing Trans Gen Biotech Co., Ltd., Beijing, China) following the manufacturer’s instructions. DNA concentration was determined by a NanoDrop 2000 UV–Vis spectrophotometer (Thermo Scientifc, Wilmington, DE, USA).

### 2.2. Design of Species-Specific Primers

Primers were designed by targeting mitochondrial DNA sequences based on both high divergence and conservation within the species. As shown in Table 1, sequences of 16S rRNA gene of turkey (GenBank Accession No. EF153719.1), 16S rRNA gene of goose (KJ124555.1), cytochrome *c* oxidase subunit I gene of pig (KJ746666.1), cytochrome *c* oxidase subunit I gene of sheep (KP702285.1), 16S rRNA gene of cattle (MN714195.1), cytochrome *b* gene of chicken (MK163565.1) and 12S rRNA gene of duck (MK770342.1) were obtained from the National Centre of Biotechnology Information (NCBI) database. Next, the MEGA6 alignment tool was employed for identifying the conservative and variable regions. Using Oligo 7.0 and BLAST programs (www.ncbi.nlm.nih.gov/blast/ accessed on 1 April 2021), primers were newly designed according to their physical parameters, such as melting temperature, secondary structures, self-complementarity and cross-reactivity. The primer pairs were synthesized by Shanghai Sangon Biological Engineering Technology & Services Co., Ltd. (Shanghai, China). To determine the mismatch between the target and nontarget species, each set of primers was in silico screened with 13 land animals: turkey (*Meleagris gallopavo*), goose (*Anser cygnoides*), pig (*Sus scrofa*), cattle (*Bos taurus*), sheep (*Ovis aries*), chicken (*Gallus gallus*), duck (*Anas platyrhynchos*), horse (*Equus caballus*), camel (*Camelus bactrianus*), ostrich (*Struthio camelus*), dog (*Canis lupus*), rabbit (*Oryctolagus cuniculus*), cat (*Felis catus*) and 3 aquatic species, namely, small yellow croaker (*Larimichthys polyactis*), tuna (*Thunnus orientalis*) and black carp (*Mylopharyngodon piceus*), using ClustalW software. The final specificity of each primer pair was examined through PCR assays against templates of all species mentioned above.

### 2.3. Simplex and Multiplex PCR

To develop a multiplex PCR method, simplex PCR was firstly carried out for each of the target species with their own primers to ensure that each target was amplified. PCR amplification (in one reaction of 25 µL, including 2.5 μL of 10 × EasyTaq^®^ Buffer, 2 μL of 2.5 mM dNTPs, 0.5 μL of EasyTaq DNA Polymerase, 0.5 μL of 10 μM each primer, and 0.01–10 ng genomic DNA of each species) was achieved using EasyTaq^®^ DNA Polymerase kit (TransGen Biotech Co., Ltd., Beijing, China). The reaction was initiated by a 5 min denaturation at 94 °C, followed by 34 cycles of denaturation at 94 °C for 30 s, annealing at 63 °C for 30 s, elongation at 72 °C for 45 s and a final elongation at 72 °C for 5 min. For universal primer pairs, the annealing was set at 56 °C. After simplex PCR assays for each species was achieved, a septuple PCR assay was developed. The 4% agarose gels were visualized and subsequently photographed in a Bio-rad GelDoc 1000 gel documentation system.

### 2.4. Test of Primers’ Specificity, Sensitivity and Reproducibility

The specificity of species-specific primers was corroborated by using template DNA isolated from all species (turkey, goose, pig, cattle, sheep, chicken, duck, horse, camel, ostrich, dog, rabbit, cat, small yellow croaker, tuna, black carp). In the preliminary phase of this experiment, simplex and septuple PCRs were respectively performed by using the DNA extracted from raw animal species. The PCR product was run on agarose gel and then visualized for the proper amplification.

The sensitivity of septuple PCR assay was confirmed by serial dilutions of the premixed DNA templates of all target species indicated, starting with 10 ng and progressing downward in one reaction. Seven concentrations (10 ng to 0.01 ng) of the target templates were used for PCR amplification determining the minimal concentration detected. PCR fragments were run on 4% agarose gel to confirm the limit of detection.

To assess the reproducibility, template DNA isolated from raw, boiled (97–99 °C, 30 min), autoclaved (121 °C, 150 kPa for 15 min) and microwave-cooked (750 W, 10 min, 119–121 °C) meat samples were individually analyzed by using the simplex PCR. The PCR product was run on agarose gel.

### 2.5. Commercial Samples

Using the present multiplex PCR method, 60 raw or thermally processed meat products including meat balls (15), meat slices (13), kebab (10), sausages (5), cutlets (10), jerky (2) and breast (5) were purchased from retail markets and supermarkets in Ningbo City, PR China, as well as online supermarket platforms, which were used for assessing the authentication of meat species. Details of the samples are listed in Table 2. All samples of meat balls, slices, kebabs, sausages, cutlets and breasts were raw materials with mechanical processing but not heat treatment, while two jerky samples, within turkey, were subjected to heat processing.

## 3. Results

### 3.1. Specificity Assays of Simplex PCR

**Figure 1:**
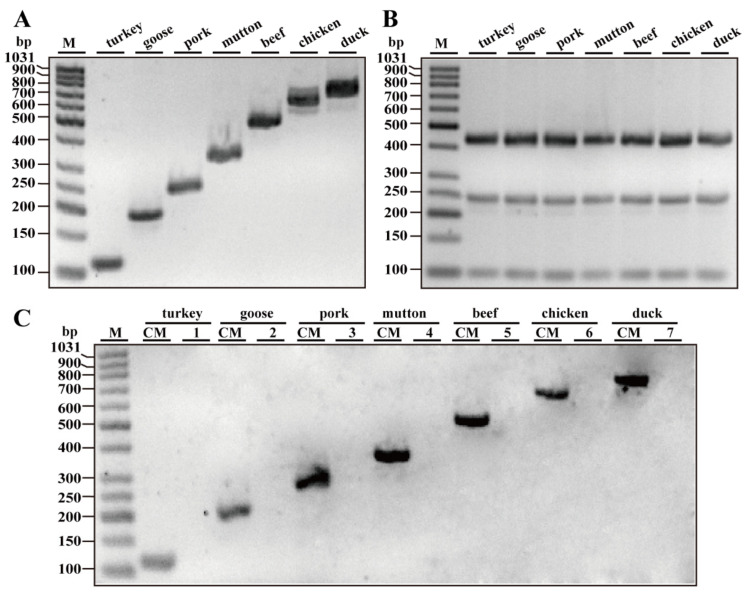
Specificity assays of simplex PCR. (**A**) Gel image of the products generated by PCR amplification with species-specific primers for turkey, goose, pig, cattle, sheep, chicken and duck using corresponding genomic DNA as a template, respectively. (**B**) As positive controls, gel image of the PCR products generated after amplification with premixed universal eukaryotic primer pairs of 12S rRNA, 16S rRNA and 18S rRNA genes for all meat species. (**C**) Gel image of the products through simplex PCR amplification using species-specific primers for turkey, goose, pig, cattle, sheep, chicken and duck. CM, a complete mixture of turkey, goose, pig, cattle, sheep, chicken and duck; 1–7, a complete DNA mixture except target species DNA. Lane M is ladder DNA.

To determine the species-specific primers, we designed many pairs of primers for each species as candidates by using Oligo 7.0 and BLAST programs. Each set of primers was compared against 16 species (turkey, goose, pig, cattle, sheep, chicken, duck, horse, camel, ostrich, dog, rabbit, cat, small yellow croaker, tuna and black carp) by simplex PCR assays (data not shown). Through gel electrophoresis, we ultimately selected the primer pairs for each species in Table 1. PCR fragments showed distinguishable bands with the predicted size of 110, 194, 254, 329, 473, 612 and 718 bp for turkey, goose, pig, sheep, beef, chicken and duck species, respectively (Figure 1A). Three pairs of universal eukaryotic primers, which target 12S rRNA, 16S rRNA and 18S rRNA genes with individual 456, 240 and 99 bp PCR fragments in all meat species, were used as positive controls for ensuring the quality of template DNAs in one PCR reaction (Figure 1B). To further test the efficiency and specificity of primers, simplex PCRs were carried out using a DNA mixture of all seven meat species. In these experiments, each set of species-specific primers yielded the expected PCR fragment by using only the template DNA mixture of seven meat species, but not with nontarget species (Figure 1C), further confirming that the new primers were highly specific for the target species.

### 3.2. Sensitivity Assays of Septuple PCR

**Figure 2:**
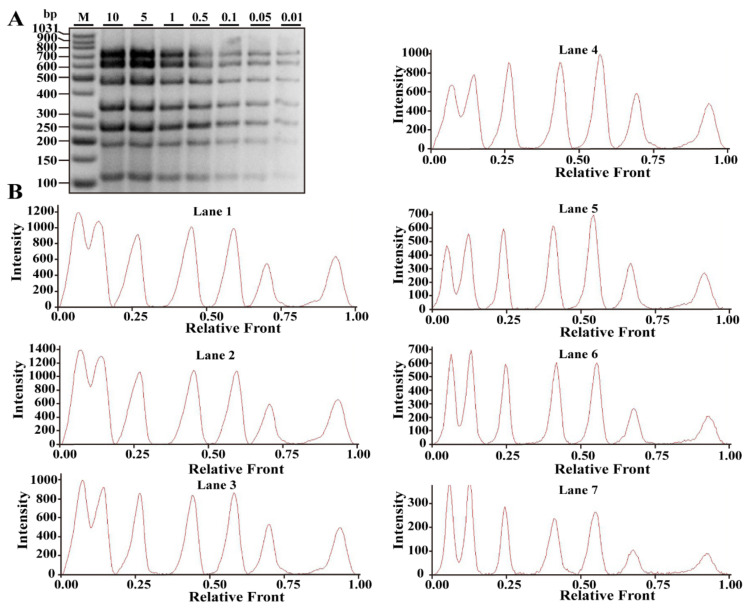
Sensitivity of the developed septuple PCR assay. (**A**) Gel image of the products generated after multiplex PCR amplifications of premixed DNA templates of all species (10, 5, 1, 0.5, 0.1, 0.05, 0.01 ng) with primers of seven meat species mixtures including turkey, goose, pig, cattle, sheep, chicken and duck. (**B**) The corresponding electropherograms of gel image (**A**). Lanes 1–7 are presented with labels (10, 5, 1, 0.5, 0.1, 0.05, 0.01) in (**A**). Lane M is ladder DNA.

A septuple PCR system was constructed by using seven sets of species-specific primers. To validate the sensitivity of the multiplex PCR assay, extracted DNA of each target species was serially diluted (10, 5, 1, 0.5, 0.1, 0.05 and 0.01 ng). PCR products were subsequently run on an agarose gel to assess the sensitivity. As can be seen from Figure 2A, the expected bands of seven meat species were obtained by multiplex PCR under the conditions of all tested concentrations (10–0.01 ng). In accordance with that of gel-view, each electropherogram clearly represented seven peaks corresponding to the seven different bands displayed in the gel-view (Figure 2B). Both intensities of bands and peaks were dramatically decreased in a concentration-dependent manner. However, even at the concentration of 0.01 ng per reaction, some PCR products of meat species can be clearly recognized in Figure 2A,B. Thus, the limit of detection of the developed septuple PCR assay was concluded to be 0.01–0.05 ng DNA.

### 3.3. Validation of Reproducibility Assay in Thermally Processed Meat

**Figure 3:**
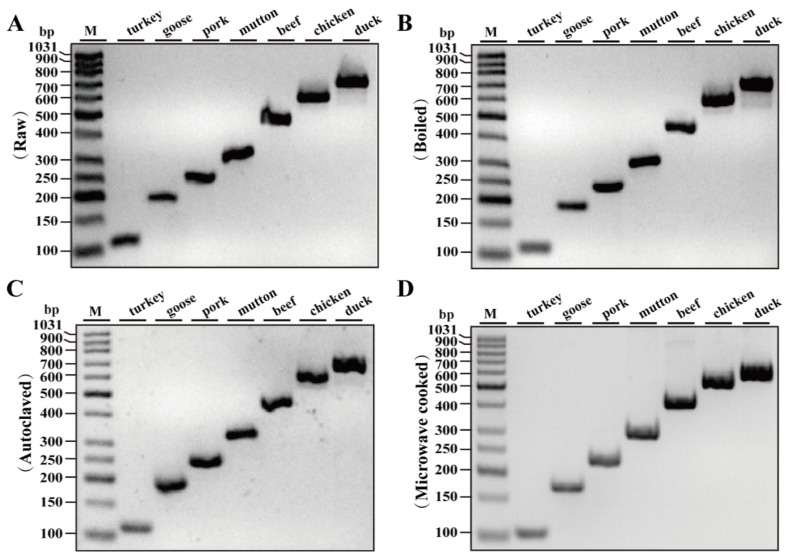
Gel image of the PCR products generated by simplex PCR amplifications with turkey, goose, pig, cattle, sheep, chicken and duck DNA extracted from raw (**A**), boiled (**B**), autoclaved (**C**) and microwave-cooked meat samples (**D**) using each species-specific primer pair. Lane M is ladder DNA.

To assess the efficiency of designed primers in detecting thermally processed meat, three different heat treatment processes were selected to treat raw meat samples as described in Section 2.4. The quality of template DNA extracted from processed meat samples was examined by simplex PCR assays. As shown in Figure 3A–D, using DNA extracted from raw, boiled, autoclaved and microwave-cooked meat samples, PCR amplification of turkey, goose, pig, sheep, beef, chicken and duck species generated the expected PCR products with 100% accuracy in meat authentication, indicating that our designed primers can be successfully employed for authenticating animal species in processed meat products.

### 3.4. Application of Multiplex PCR Assay on Commercially Processed Meat Products

**Figure 4:**
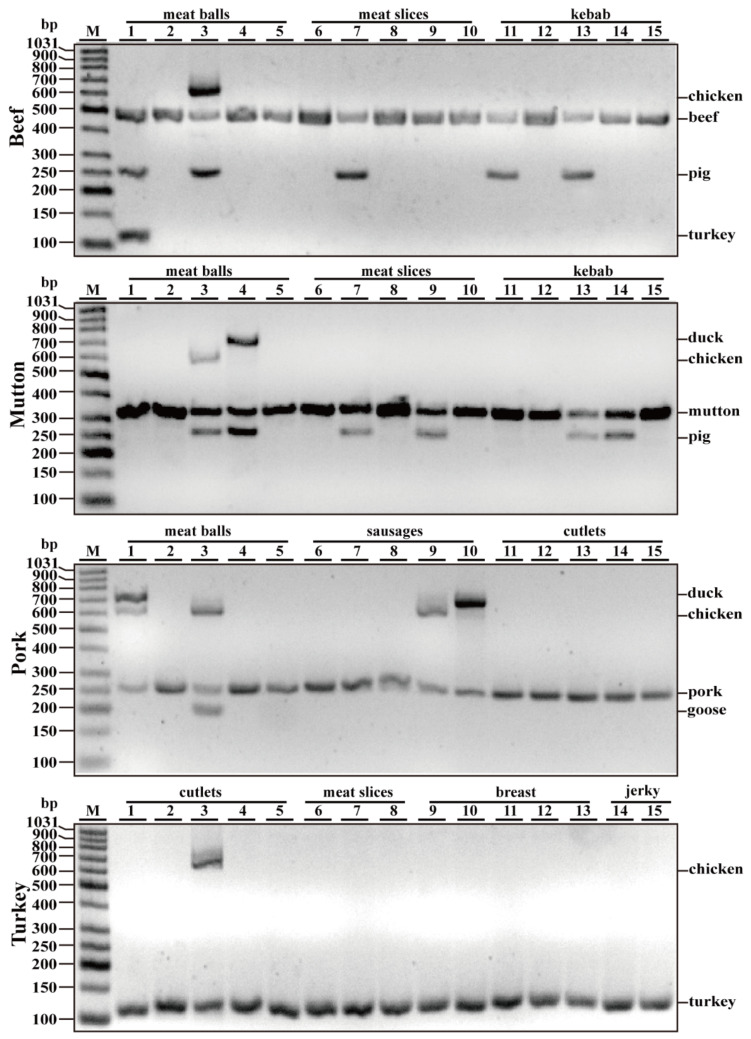
Analysis of commercial foodstuffs using the developed septuple assay. Gel image of the fragments generated by multiplex PCR amplifications using DNA obtained from commercial meat products with premixed primers for seven meat species including turkey, goose, pig, cattle, sheep, chicken and duck.

The real-world food products were examined using the developed septuple PCR. The survey was conducted with 60 commercial samples of beef, mutton, pork and turkey (15 samples each). As shown in Figure 4 and summarized in Table 2, most of the samples had the same ingredients as labeled, without contamination. However, 5 of 15 (33.3%) beef samples, 6 of 15 (40.0%) mutton samples, 4 of 15 (26.7%) pork samples and 1 of 15 (6.7%) turkey samples contained some unlisted meat species. The survey revealed that inexpensive chicken, duck and pork meats were frequently adulterated products. The results further corroborated the efficiency of the developed septuple PCR assay in identification of commonly consumed meats.

## 4. Discussion

Frequent meat frauds have aroused significant social attention because adulteration risks food safety, breaches market rules and even threatens public health [8]. In recent years, adulteration practice has been ingeniously applied to treated meat products showing similar morphological and physical appearance to pure meat. Nowadays, PCR-based techniques are the effective methods for species authentication. Real-time PCR techniques and microchip electrophoresis-dependent multiplex PCR methods require special infrastructures [11,29,30]; multiplex PCR assays through simple agarose gel analysis minimizes the cost drastically on a large scale and can be easily carried out to verify the identity of ingredients in foodstuffs [6,31,32]. As seen in Table 3, much is known about multiplex PCR assays that simultaneously verify two to six meat ingredients in one reaction. Relatively little information is available on multiplex PCR methods for authenticating more meat species simultaneously. Although one study authenticated 10 animal species (beef, sheep, pork, chicken, turkey, cat, dog, mouse, rat, human), it was achieved by two tube multiplex assays, where every five animal species were verified by a pentaplex PCR assay in one reaction [33]. Similarly, using two tube independent pentaplex PCR assays with ten pairs of primers, 14 animal species including cattle, donkey, *Canidae* (dog, fox, raccoon-dog), deer and horse, pig, *Ovis* (sheep, goat), poultry (chicken, duck), cat and mouse were detected through chip electrophoresis; however, the multiplex PCR failed to accurately distinguish sheep and goat within Ovis, dog, fox and raccoon-dog within Canidae, and chicken and duck within poultry [11]. Therefore, there is still a lack of more efficient detection methods with low cost for supervising more meat content. The goal of the present study was to develop a multiplex PCR method for reliable and efficient identification of ruminant, poultry and pork materials.

The choice of animal species was considered based on actual adulteration cases, with a higher practicability in Chinese markets. We found that multiplex PCR with increased species-specific primers in one reaction led to more opportunities of cross-reactivity with each other, or generated unexpected bands, which may limit the availability of multiplex PCR for verifying more animal species. To provide a multiplex PCR method that detects more animal species in a single assay platform, we designed many sets of primers throughout target mitochondrial DNA sequences such as cytochrome b gene, D-loop, 12S and 16S rRNA genes, ATPase subunits 8 and 6 genes and NADH dehydrogenase genes using Oligo 7.0 and Primer-BLAST programs. Through screening species-specific primer pairs, a species-specific septuple PCR method was ultimately developed and optimized to simultaneously detect turkey (110 bp), goose (194 bp), pig (254 bp), sheep (329 bp), beef (473 bp), chicken (612 bp) and duck (718 bp) in one reaction. To ensure the quality of template genomic DNA in one PCR reaction, a universal eukaryotic primer set that amplifies a bigger PCR fragment than that of all meat species tested should be chosen as the preferred positive control. However, to our knowledge, little information is available on a universal eukaryotic primer set amplifying the fragment with more than 700 bp length. As alternatives, we chose three pairs of universal eukaryotic primers, which target different mitochondrial DNA sequences, including 12S rRNA, 16S rRNA, 18S rRNA genes, and amplifies distinguishable 456, 240 and 99 bp PCR fragments in all meat species, respectively [26,27,28]. In addition, the control primer set should be inserted in the multiplex assay; these PCR fragments were too close to that of turkey (110 bp), pig (254 bp) and beef (473 bp) to discriminate each other. Accordingly, these universal primer pairs were used in a single PCR in this study. Figure 1B shows the expected bands of each primer set in all meat species, implying the high quality of genomic DNA used in this study.

Through the specificity test, we validated that the primers were highly specific to each of particular species and had no cross-reactivity, at least with the 15 animal species tested. The detection limit of this particular assay on reference meat samples was 0.01–0.05 ng, indicating that this method is highly sensitive and reliable. Using the DNA isolated from raw, boiled, autoclaved and microwave-cooked samples of seven meat species, simplex PCR assays generated all expected PCR products, suggesting that PCR assay with our primers had a high reproducibility in processed meat samples. Most importantly, multiplex PCR assay on commercially available processed meat products revealed that inexpensive chicken, duck and pork meats were adulterated products (Table 2 and Figure 4). Consistent with previous reports, inexpensive poultry meat readily evades visual detection and is frequently adulterated into other meat products, in particular, processed beef, mutton and pork [41,42,43]. Perhaps, economically driven thoughts of manufacturers or peddlers are a critical factor for the replacement of expensive, high-quality meat with inferior and low-cost ones. Collectively, the developed septuple PCR assay is not only reliable and efficient but is also a sensitive detection method for the identification of meat species in actual adulteration events. However, vegetable proteins such as soybean are found to a substitute ingredient for muscle proteins, due to their low cost of production [8,38,44]. In addition, some surveys demonstrate that inexpensive fish species are adulterated into meat products [44,45]. Therefore, we still cannot exclude the possibility that vegetable proteins and fish sources may be present in commercial meat products. Considering the fact that multiplex PCR with increased species-specific primers in one reaction may cause more opportunities of cross-reactivity with each other, or generated unexpected bands, a more efficient method for identification of meat adulteration should be developed in future study.

## 5. Conclusions

The aim of this study is to provide reliable adulteration detection, by means of septuple PCR, which can simultaneously authenticate seven animal species of turkey, goose, pig, sheep, beef, chicken and duck. The assay is also quite sensitive to enable detection of 0.01–0.05 ng DNA templates for each species per reaction, thus making it qualified for authenticating meat species in commercial, real-world foodstuffs. By simple agarose gel analysis, without expensive equipment or a high level of technical skill, this septuple PCR method could be more broadly used for detecting sources of meat species in foodstuffs in which adulteration is suspected.

## Figures and Tables

**Table 1:** Oligonucleotide primers for meat species used in this study.

Primers	Genes	Sequence (5′–3′ Direction)	Amplicons (bp)	Reference or Source
Turkey	16S rRNA	CTCTAGCCCAACCACCCAT	110	this study
		GCGCCTAAGGTCTTTTCTATCAC		
Goose	16S rRNA	TTAGACGCGATAGAGACCCCA	194	this study
		GTTCGCTCTCTTTAACTGCTTG		
Pig	cytochrome c oxidase subunit I	CAGCCCGGAACCCTACTTG	254	this study
		GTTCATCCAGTACCCGCTCC		
Sheep	cytochrome c oxidase subunit I	AGATATCGGCACCCTTTACCTTC	329	this study
		CTGCTCCGGCCTCAACCAT		
Beef	16S rRNA	GTGCCTGATAATACTCTGACCAC	473	this study
		CACCCCAACCGAAACTACCAA		
Chicken	cytochrome b	TTTCGGCTCCCTATTAGCAGTC	612	this study
		AGTATGAGAGTTAAGCCCAGA		
Duck	12S rRNA	TGCCCTCAATAGCCTTCACC	718	this study
		CATACTTCTTTCCGTGTTGCC		
Eukaryotes	12S rRNA	CAACTGGGATTAGATACCCCACTAT	456	[26]
		GAGGGTGACGGGCGGTGTGT		
Eukaryotes	16S rRNA	AAGACGAGAAGACCCTATGGA	240	[27]
		GATTGCGCTGTTATCCCTAGGGTA		
Eukaryotes	18S rRNA	AGGATCCATTGGAGGGCAAGT	99	[28]
		TCCAACTACGAGCTTTTTAACTGCA		

**Table 2:** Results of multiplex PCR assay performed on commercial meat products.

Products	No	Labelled	Detected Species							Adulteration
			Turkey	Goose	Pig	Sheep	Beef	Chicken	Duck	
Beef	15									5 (33.3%)
meat balls	5	beef	1/5 ^b^	—	1/5 ^a^, 1/5 ^b^	—	5/5	1/5 ^a^	—	
meat slices	5	beef	—	—	1/5	—	5/5	—	—	
kebab	5	beef		—	2/5	—	5/5	—	—	
Mutton	15									6 (40.0%)
meat balls	5	mutton	—	—	1/5 ^a^, 1/5 ^b^	5/5	—	1/5 ^a^	1/5 ^b^	
meat slices	5	mutton	—	—	2/5	5/5	—	—	—	
kebab	5	mutton	—	—	2/5	5/5	—	—	—	
Pork	15									4 (26.7%)
meat balls	5	pig	—	1/5 ^b^	5/5	—	—	1/5 ^a^, 1/5 ^b^	1/5 ^a^	
sausages	5	pig	—	—	5/5	—	—	1/5 ^a^	1/5 ^b^	
cutlets	5	pig	—	—	5/5	—	—	—	—	
Turkey	15									
cutlets	5	turkey	5/5	—	—	—	—	1/5	—	1 (6.7%)
meat slices	3	turkey	3/3	—	—	—	—	—	—	
breast	5	turkey	5/5	—	—	—	—	—	—	
jerky	2	turkey	2/2	—	—	—	—	—	—	

A horizontal line (—) denotes no PCR product detected. In each row, the meat samples labeled with same letter (^a^ or ^b^) represent the identical meat samples, while different letters indicate a difference in meat samples.

**Table 3:** Comparative analysis of recently published multiplex PCR assays for the identification of meat species.

Multiplex PCR Type	Sp. No ^a^	Detection Items	Detection Limit	Detection Method ^b^	Reference or Source
Septuple	7	turkey, goose, pig, sheep, beef, chicken, duck	0.01–0.05 ng DNA	Gel	This study
Multiplex	4	ruminant, poultry, pork, and donkey	0.01–0.1 ng/μL DNA	Gel	[25]
Hexaplex	6	chicken, cow/buffalo, sheep/goat, horse/donkey, pork, dog	0.03–0.05 ng DNA	Gel	[31]
Multiplex	5	sheep/goat, bovine, chicken, duck, pig	0.5 ng DNA	Gel	[6]
Multiplex	2	cattle, buffalo	2.23–2.31 ng/μL DNA	Gel	[34]
Quadruple	4	fox, mink, or raccoon in beef and mutton	1% for each species	Gel	[35]
Pentaplex	5	dog, duck, buffalo, goat, sheep	0.1–0.32 ng DNA	Gel	[21]
Multiplex (two-tube)	14	cattle, donkey, Canidae (dog, fox, raccoon-dog), deer and horse, pig, Ovis (sheep, goat), poultry (chicken, duck), cat, mouse	0.02–0.2 ng DNA	Chip	[11]
Quadruplex	4	chicken, mutton, beef, pork	16 pg DNA, 0.01% of each species	Gel	[36]
Multiplex (two-tube)	10	beef, sheep, pork, chicken, turkey, cat, dog, mouse, rat, human	30 pg DNA	Gel	[33]
Tetraplex	3	pig, cattle, fish, eukaryotic18S rRNA	0.001–0.1 ng DNA	Gel	[37]
Hexaplex	6	horse, soybean, sheep, poultry, pork, cow	0.01% for each species	Gel	[38]
Octuplex	8	dog, chicken, cattle, pig, horse, donkey, fox, and rabbit	0.05 ng/μL DNA	Gel	[27]
Multiplex	3	chicken, duck and goose	0.05 ng DNA, 1% for each species	Gel	[39]
Multiplex	5	cat, dog, pig, monkey, rat	0.01–0.02 ng DNA	Chip	[24]
Quadruple	4	beef, pork, mutton, duck	0.1 ng DNA	Gel	[40]

^a^ Species number; ^b^ Chip, microchip electrophoresis; Gel, agarose gel electrophoresis.

## Data Availability

Not applicable.
